# Pretreatment With *Bacillus cereus* Preserves Against D-Galactosamine-Induced Liver Injury in a Rat Model

**DOI:** 10.3389/fmicb.2019.01751

**Published:** 2019-07-31

**Authors:** Ya-Ting Li, Jian-Zhong Ye, Long-Xian Lv, Hong Xu, Li-Ya Yang, Xian-Wan Jiang, Wen-Rui Wu, Ding Shi, Dai-Qiong Fang, Xiao-Yuan Bian, Kai-Cen Wang, Qiang-Qiang Wang, Jiao-Jiao Xie, Yan-Meng Lu, Lan-Juan Li

**Affiliations:** ^1^State Key Laboratory for the Diagnosis and Treatment of Infectious Diseases, The First Affiliated Hospital, School of Medicine, Zhejiang University, Hangzhou, China; ^2^Collaborative Innovation Center for the Diagnosis and Treatment of Infectious Diseases, Hangzhou, China; ^3^Department of Orthopedics, Xiaoshan Traditional Chinese Medical Hospital, Hangzhou, China

**Keywords:** gut microbiota, Bacillus cereus (B. cereus), D-galactosamine (D-GalN), probiotic, acute liver injure

## Abstract

*Bacillus cereus* (*B. cereus*) functions as a probiotic in animals, but the underlying mechanisms remain unclear. We aim to evaluate the protective effects and definite mechanism by which orally administered *B. cereus* prevents D-galactosamine (D-GalN)-induced liver injury in rats. Twenty-one Sprague–Dawley rats were equally assigned into three groups (*N* = 7 animals per group). *B. cereus* ATCC11778 (2 × 10^9^ colony-forming units/ml) was administered to the *B. cereus* group via gavage, and phosphate-buffered saline was administered to the positive control (PC) and negative control (NC) groups for 2 weeks. The PC and *B. cereus* groups received 1.1 g/kg D-GalN via an intraperitoneal injection to induce liver injury. The blood, terminal ileum, liver, kidney and mesenteric lymph nodes (MLNs) were collected for histological examinations and to evaluate bacterial translocation. Liver function was also determined. Fecal samples were collected for deep sequencing of the 16S rRNA on an Illumina MiSeq platform. *B. cereus* significantly attenuated D-GalN-induced liver injury and improved serum alanine aminotransferase (ALT) and serum cholinesterase levels (*P* < 0.05 and *P* < 0.01, respectively). *B. cereus* modulated cytokine secretion, as indicated by the elevated levels of the anti-inflammatory cytokine interleukin-10 (IL-10) in both the liver and plasma (*P* < 0.05 and *P* < 0.01, respectively) and the substantially decreased levels of the cytokine IL-13 in the liver (*P* < 0.05). Pretreatment with *B. cereus* attenuated anoxygenic bacterial translocation in the veins (*P* < 0.05) and liver (*P* < 0.05) and upregulated the expression of the tight junction protein 1. The gut microbiota from the *B. cereus* group clustered separately from that of the PC group, with an increase in species of the *Ruminococcaceae* and *Peptococcaceae* families and a decrease in those of the *Parabacteroides*, *Paraprevotella*, and *Desulfovibrio* families. The potential probiotic *B. cereus* attenuated liver injury by restoring the gut flora balance and enhancing the intestinal barrier function.

## Introduction

Most strains characterized as lactic acid bacteria, including *Bifidobacterium* sp., *Lactobacillus* sp., and *Enterococcus* sp., are proposed to function as probiotics according to the following definition: “living microorganisms exert health benefits beyond inherent basic nutrition” (Ljungh and Wadstrom, 2006).

Numerous *Bacillus cereus* (*B. cereus*) subspecies have been used as probiotics in animals, including *B. cereus* (2), *B. cereus var. toyoi* (ToyocerinÔ, Rubinum, Rubí, Spain), and *B. cereus IP 5832* (BactisubtilÔ, Marion Merrell S.A. Bourgoin-Jallieu, France or PaciflorÔ, Hoechst Roussel Vet GMBH, Wiesbaden, Germany) (Trapecar et al., 2011). The beneficial effects of probiotic *B. cereus* strains used as feed supplements on the health of animals are generally acknowledged (Altmeyer et al., 2014).

A number of probiotic food supplements and therapeutic products containing or consisting of *Bacillus* strains/species have been available for human consumption during the past 2 decades (Hatziloukas and Panopoulos, 1992). Some *Bacillus* species have been explored for use as pharmaceutical preparations or probiotics for humans, such as *Bacillus licheniformis*, *Bacillus coagulans*, *Bacillus subtilis*, and *B. cereus* (Urdaci et al., 2004). Moreover, *B. cereus*, whose national medicine permission number (NMPN) is s10980014, has already been licensed as a probiotic in China.

D-galactosamine (D-GalN)-induced acute liver injury is used as an established experimental model to study liver disease (Lu et al., 2014). Uridine triphosphate deficiency is caused by D-GalN and results in liver apoptosis and necrosis, metabolic changes and inhibition of the synthesis of RNA and proteins (Wang et al., 2014; Yang et al., 2014).

Accumulating evidence indicates an adaptive commensal relationship between the progression of hepatic injury and the gut microbiota (Festi et al., 2006; Tranah et al., 2013). Disrupted hepatic pathology and physiology alter the intestinal barrier structure, allowing the penetration of microbes or bacterial products and macromolecules, such as lipopolysaccharide (LPS), from the intestines to the liver. The gut flora and bacterial translocation (BT) enhance the propagation of inflammation, tissue damage, sepsis and spontaneous bacterial peritonitis in patients with complications of liver diseases (Wiest and Garcia-Tsao, 2005). The production of the inflammatory cytokines interleukin-1α/β (IL-1α/β), tumor necrosis factor-α (TNF-α), CC-chemokine ligand 2 (CCL2), and IL-6 is stimulated when cytokines are recruited in response to liver injury (Gehrke et al., 2018).

Modulation of the immune system induced by increased expression of the cytokines TNF-α and interferon-γ (IFN-γ) has been proposed to be a mechanism underlying the functions of probiotics (Braat et al., 2004), and *B. cereus var. toyoi* has been shown to enhance systemic immune responses by increasing the percentage of CD8^+^ lymphocytes in the jejunal epithelium (Scharek et al., 2007; Schierack et al., 2007). Furthermore, among 14 *Bacillus* strains isolated from commercial probiotic products, *B. cereus* displayed the best adhesion to intestinal surfaces, which is an important characteristic of a probiotic (Sanchez et al., 2009). Moreover, *B. cereus* consumes oxygen in the intestine, providing a local anaerobic environment that aids in the growth of profitable bacteria (Wang et al., 2004). Mixing *B. cereus* with *Bifidobacterium* reduces BT in scalded rats further than that achieved with *Bifidobacterium* alone, and the intestinal flora, mucosal structure and local immunity are significantly improved (Wang et al., 2004). Manipulations of the gut microbiota designed to reinforce gut barrier function and suppress BT could exert protective effects on hepatic injury (Seki and Schwabe, 2015).

The studies listed above have confirmed the probiotic role of *B. cereus* in animals, but the underlying mechanisms remain unclear. While the potential beneficial effects of *B. cereus* on a weakened gut barrier are encouraging (Wang et al., 2004), the ability of *B. cereus* to increase intestinal barrier function by disrupting liver physiology and modulating the gut microbiota has not yet been determined. This study aims to explore the protective effect of pretreatment with *B. cereus* on hepatic damage induced by D-GalN in rats and the underlying mechanisms.

## Materials and Methods

### Strain and Culture Conditions

*B. cereus (* ATCC strain 11778) was purchased from the American Type Culture Collection (Rockville, MD, United States) and grown at 37°C under aerobic conditions in brain heart infusion (BHI) (QingDao RiShui, Ltd., Qingdao, China) broth for 24 h. Cells were washed with sterile phosphate buffer and then harvested at a concentration of 3 × 10^9^ CFU/ml by centrifugation at 8,000 × *g* for 10 min. The same solution was used to resuspend cells, which were stored at 4°C until further use.

### Rat Model of Acute Liver Injury

This study was carried out in accordance with the principles of the Basel Declaration and recommendations of the “Guidelines for Experimental Animals,” Ministry of Science and Technology (Beijing, China). The protocol was approved by the Animal Care Committee of Zhejiang University School of Medicine (permit number: 2017-591). Twenty-one male specific pathogen-free (SPF) Sprague–Dawley rats (8 w, 258.13 ± 7.068 g) were equally divided into 3 groups with randomized blocks of 7 replicates as follows: the negative control (NC) group received oral administrations of phosphate buffer saline and an intraperitoneal injection of normal saline; the positive control (PC) group received oral administrations of phosphate buffer saline and an intraperitoneal injection of D-GalN; the *B. cereus* ATCC 11778 group received oral administrations of *B. cereus* ATCC 11778 and an intraperitoneal injection of D-GalN. In accordance with ethical requirements, all rats were fed standard chow and housed at 22°C with a controlled 12 h light/12 h dark cycle. One milliliter of normal saline or a freshly prepared bacterial suspension (at 3 × 10^9^ CFU/ml) was administered to the NC, PC, and *B. cereus* groups via gavage once daily for 2 weeks.

D-GalN, produced by Sigma (St. Louis, MO, United States), was dissolved in normal saline and administered to rats in the PC and *B. cereus* groups via an intraperitoneal injection on the 15th day. The rats were sacrificed aseptically 24 h after the D-GalN challenge after being anesthetized with 4% chloral hydrate. Atropine was applied to inhibit respiratory secretions at a dose of 1 mg/100 ml solution.

### Biochemical Indicators of Liver Function

Venous blood samples were centrifuged at 3,000 × *g* for 10 min at 4°C to extract the serum for further analysis. Standard methods were employed to assess the serum levels of alanine aminotransferase (ALT), aspartate aminotransferase (AST), γ-glutamyltransferase (GGT), globulin and total bilirubin (TBil), glycylproline dipeptidyl aminopeptidase (GPDA), total bile acid (TBA), cholinesterase and albumin using a Hitachi 7600–210 automatic analyzer (Hitachi, Tokyo, Japan) according to the manufacturer’s instructions (Cho et al., 2014).

### Plasma Cytokine Analysis

Before analysis, venous blood samples were centrifuged at 3,000 × *g* for 10 min at 4°C, and the plasma (20 μl) was stored at –80°C. According to the manufacturer’s instruction, the Bio-Plex Pro Rat Cytokine Plex Panel (Hercules, CA, United States) was used to measure cytokine concentrations, which were analyzed using Bio-Plex Manager 6.1 software (Bio-Rad) and the MAGPIX system (Luminex Corporation).

Plasma cytokine levels were assessed using the Bio-Plex Pro Rat Cytokine magnetic bead suspension array (Bio-Rad, CA, United States), according to the manufacturer’s instructions. The targeted cytokines included interleukin (IL)-1α; IL-1β; IL-2; IL-4; IL-5; IL-6; IL-7; IL-10; IL-12 (p70); IL-17A; IL-18; erythropoietin; granulocyte-macrophage colony-stimulating factor; tumor necrosis factor (TNF)-α; granulocyte colony-stimulating factor; vascular endothelial growth factor (VEGF); growth-regulated oncogene-keratinocyte chemoattractant; interferon (IFN)-γ; regulated on activation, normal T cell expressed and secreted (RANTES); granulocyte-macrophage colony-stimulating factor (G-CSF); granulocyte-macrophage colony-stimulating factor (GM-CSF); and macrophage colony-stimulating factor (M-CSF). The Bio-Plex 200 analyzer was used to examine the samples, and Bio-Plex Manager 6.0 software was used to calculate the results (Rotstein, 2014).

### Bacterial Translocation (BT)

A piece of the left hepatic lobe, mesenteric lymph nodes (MLNs) and kidney samples were aseptically removed and weighed. Autoclaved glass homogenizers were used to mill the tissue homogenates diluted with saline, which were separately incubated in duplicate at 37°C for 48 h under aerobic and anaerobic conditions on BHI agar (Oxoid, Thermo Fisher Biochemicals Ltd., Beijing, China). The BT percentages in every group were recorded as fractions and percentages.

### Analysis of Histopathological Staining

The terminal ileum and left lobe of hepatic specimens were collected from anesthetized rats, immediately fixed with 10% neutral buffered formalin and embedded in paraffin to evaluate the histology. Two-micrometer-thick sections of paraffin-embedded samples were cut and stained with hematoxylin and eosin (H&E). At least three slides from each specimen were analyzed in a blinded manner.

Two categories of the Histological Activity Index (Knodell et al., 1981) were used to semiquantitatively assess liver tissue damage, the inflammation of the portal region (score, 0–1 or 3–4 points) and focal necrosis and intralobular degeneration (score, 0–1 or 3–4 points) (Chiu et al., 1970). The classification of intestinal mucosal lesions is described below. A score of 0 is defined as normal mucosal villi, and an extension of the subepithelial Gruenhagen’s space is marked as a score of 1. The expansion of space in the tissue receives a score of 2. A score of 3 is defined as a massive epithelial lifting down the side of a villus. In samples receiving a score of 4, the villus is denuded of the epithelium. The loss of the lamina propria is characterized as a score of 5.

### Transmission Electron Microscopy

The ileal mucosal samples were postfixed with a 2.5% glutaraldehyde solution, dehydrated and embedded in epoxy resin. Uranyl acetate and lead citrate were applied to stain ultrathin sections (Dias et al., 2017). The ultrastructure of the ileal mucosa, including the linear density, diameter and length of microvilli, was analyzed using a Tecnai 10 electron microscope (Philips, Eindhoven, The Netherlands) (Brown, 1962).

### Microbial Community and PICRUSt Analyses

The microbiota analysis was conducted with fecal samples collected from the cecum. The cecum is an anatomically distinct structure located between the ileum and colon. Sufficient quantities of microbiota were colonized for microbiota analysis and back-up (Turnbaugh et al., 2006). Total genomic DNA was extracted using a QIAamp^®^ Fast DNA Stool Mini Kit (QIAGEN, Hilden, Germany) according to the manufacturer’s handbook. A NanoDrop spectrophotometer and agarose gel electrophoresis were separately employed to verify the quantity and quality of DNA. A 1 ng/μl solution of the extracted DNA was stored at –20°C until further use. The diluted DNA was used as a template for the PCR amplification of bacterial 16S rRNA genes with the HiFi Hot Start Ready Mix (KAPA) and barcoded primers. For the bacterial diversity analysis, the V3–V4 variable regions of the 16S rDNA gene were amplified by PCR using the primers 343F and 798R. The quality of the amplicon was determined using gel electrophoresis. The amplified product was purified with AMPure XP beads (Agencourt) and then subjected to another round of amplification, and a Qubit dsDNA assay kit was used to quantify the final amplicon. The sequence was subsequently determined from a pool of equal amounts of the purified amplicon.

Raw sequencing was conducted on an Illumina MiSeq platform (Illumina, San Diego, CA, United States) according to the manufacturer’s instructions and recorded in FASTQ format. Trimmomatic software was used to preprocess paired-end reads (Bolger et al., 2014) and to detect and remove ambiguous bases (N). A sliding-window trimming procedure was applied to sequences to remove the bases with an average quality score of less than 20, and paired-end reads were assembled by QIIME software (version 1.8.0) and Trimmomatic, FLASH (Reyon et al., 2012) based on parameters of a 20% maximum mismatch rate and a 10–200 bp overlap (Caporaso et al., 2010). Noise was removed from the sequences by removing homologous and ambiguous reads. When 75% of the bases in a sequence had quality scores greater than 20, the read was retained. Finally, chimeras were detected and abandoned.

UPARSE software was used to build clean reads from the primer sequences, which were then clustered into operational taxonomic units (OTUs) with a 97% similarity cutoff (Edgar, 2013), and a representative read of each OTU was selected by QIIME. All representative reads were annotated and subjected to a BLAST search against the Greengenes (16S rDNA) or SILVA database version 123, which align with the Ribosomal Database Project (RDP) classifier and display a 70% confidence threshold (Wang et al., 2007). The metabolic functions of microbial communities were analyzed and predicted by PICRUSt v1.0.06 referencing the Kyoto Encyclopedia of Genes and Genomes (KEGG) Orthology (KO) database (Langille et al., 2013; Kanehisa et al., 2014). The biological relevance and statistical significance of differences between various taxa were determined by linear discriminant analysis (LDA) effect size (LEfSe) (Lu et al., 2016). The clustering of microbial communities was calculated using an analysis of similarities (ANOSIM) with Bray–Curtis distance matrices. The GenBank Sequence Read Archive contains the sequence data under the number PRJNA516292.

### Lipopolysaccharide Binding Protein Measurement

Blood collected from the inferior vena cava was centrifuged at 1000 × *g* for 10 min to separate the serum. The levels of LPS-binding protein (LBP) were measured using an ELISA Kit (GD-S1538-K, Shanghai, China) to indirectly reflect the endotoxin levels.

### Immunofluorescence Staining

Two-micrometer-thick sections of the paraffin-embedded terminal ileum were sliced, dewaxed, rehydrated and incubated with 3% H_2_O_2_ using standard methods. Sections were incubated with a 1:1000 dilution of anti-zonula occludens-1 (ZO-1) antibodies overnight at 4°C and then incubated with a 1:100 dilution of a FITC-conjugated goat anti-rabbit secondary antibody (Beyotime, Shanghai, China) for 1 h at room temperature. Terminal ileum sections were finally scanned using a Zeiss LSM T-PMT confocal microscope (Zeiss, Jena, Germany).

### RNA Extraction and Real-Time PCR

Briefly, using previously described standard procedures (Fang et al., 2017), total RNA was extracted from intestinal segments and hepatic samples with an RNeasy Mini Kit (Qiagen, Hilden, Germany). The integrity of the RNA was verified with ethidium bromide staining. The primer sequences are listed in Supplementary Table 1. The expression data were calculated by comparing the cycle thresholds in triplicate samples. Relative mRNA expression was normalized to the expression of β-actin and detected via the 7500 real-time PCR system (Applied Biosystems) and a One Step SYBR PrimeScript plus RT-PCR kit (Takara Biomedicals, Kusatsu, Japan).

### Statistical Analysis

One-way analysis of variance (ANOVA) was used to analyze the significance of differences among groups, and the Bonferroni’s or Dunnett’s T3 *post hoc* test was performed for pairwise comparisons as appropriate. The Chi-square test was performed to evaluate the frequency of BT between different cohorts. Correlation coefficients between variables were calculated using Spearman’s rank correlation analysis. The normally distributed values are presented as the means ± SEM. Data with a nonnormal distribution are presented as medians and interquartile ranges. All statistical analyses were conducted using GraphPad Prism and SPSS version 17.0 software (SPSS Inc., Chicago, IL, United States), and *P* < 0.05 were considered statistically significant.

## Results

### Pretreatment With *B. cereus* Improved D-GalN-Induced Liver Injury and Induced Anti-inflammatory Characteristics

Prior to the D-GalN injection, the health status of all rats was observed and evaluated in our study. After intragastric administration of *B. cereus* for 14 days, neither of weight loss, depression, diarrhea or death was observed in *B. cereus* group. Body mass were measured at baseline and every 2 days after the first gavage administration to monitor the health of the animals. No significant differences were reported among the three groups at either time point (Supplementary Figure 1). Then, after D-GalN injection, one rat in the PC group died (*n* = 6), but none of the rats in the *B. cereus* prophylaxis group (*n* = 7) or NC group (*n* = 7) died. The *B. cereus* prophylaxis significantly reduced the D-GalN-induced increase in serum ALT levels and improved serum cholinesterase levels compared to those in the PC group (which was administered PBS) (*P* < 0.05 and *P* < 0.01, respectively) (Table 1). Moreover, significant differences in ALT and cholinesterase levels were observed between the *B. cereus* group and the NC group (*P* < 0.01 for each). Additionally, peripheral blood liver function tests showed markedly increased levels of globulin (*P* < 0.05) and albumin (*P* < 0.05) in the *B. cereus* group compared to those in the PC group (Table 1).

**TABLE 1:** Effects of *B. cereus* on liver function, serum LBP levels and plasma cytokine levels.

	**NC (negative control) *N* = 7**	**PC (positive control) *N* = 6**	Bacillus cereus N = 7
ALT	48.00±10.41^b^	6208.00±441.17	4840.00±313.84^a,d^
Albumin	13.33±1.63	14.00±2.03	23.14±4.339^a^
Globulin	27.20±2.65	27.17±2.51	36.57±2.92^a^
Cholinesterase	432.00±21.12	444.00±37.0	582.00±19.74^b,d^
RANTES	188.29±69.47	1755.71±51.89	1431.00±96.99^a,d^
IL-7	790.71±72.12	1215.5±136.16	702.29±124.30^a^
IL-10	668.86±31.374	775.5±42.63	1125.5±22.053^b,d^
LBP	72.9±12.0^b^	172.1±15.4	120.99±27.1^a,c^

**Data are presented as the mean ± SEM. ^a^P < 0.05 and ^b^P < 0.01 compared with the PC group, ^c^P < 0.05 and ^d^P < 0.01 for the comparison of the B. cereus group with the NC group. NC, negative control; PC, positive control; ALT, alanine aminotransferase; RANTES, regulated on activation, normal T cell expressed and secreted; LBP, lipopolysaccharide-binding protein.**

**FIGURE 1:**
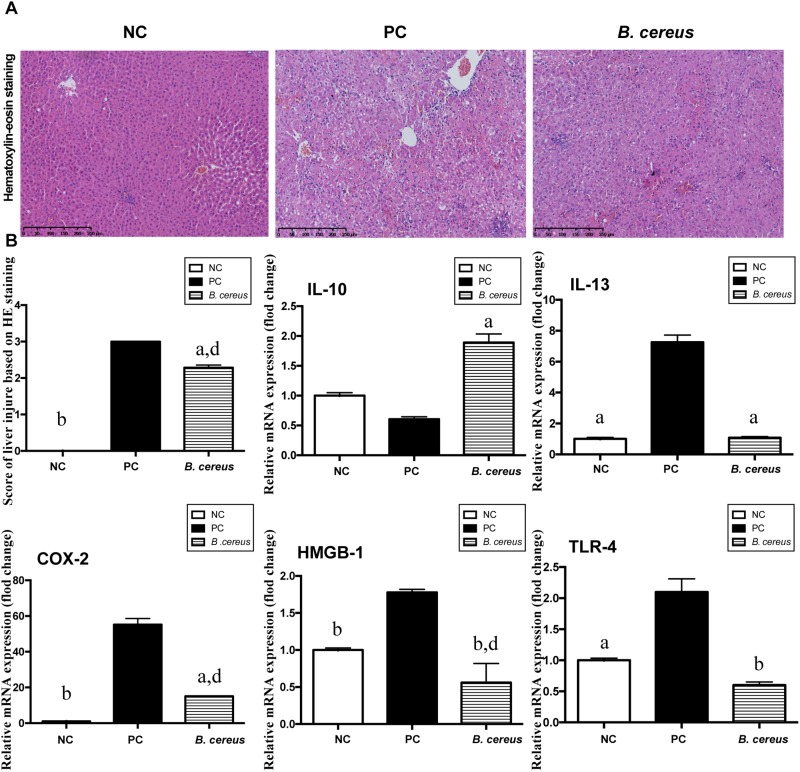
*B. cereus* alleviated D-GalN-induced liver injury. **(A)** Representative images of hepatic H&E staining. **(B)** Histological scores of liver tissues based on these images: hepatic expression of the apoptosis-related cytokines COX-2 and HMGB-1 and the inflammatory cytokines IL-10, IL-13, and TLR-4. Gene expression was determined by performing quantitative PCR analysis of the total mRNA extracted from liver fragments. The results are presented as fold changes relative to the levels in the NC group. All data are presented as the mean ± SEM compared with the PC group. (NC group, *n* = 7; PC group, *n* = 6; *B. cereus* group, *n* = 7). ^a^*P <* 0.05 and ^b^*P <* 0.01 compared with the PC group, ^d^*P <* 0.01 for the comparison of the *B. cereus* group with the NC group. NC, negative control; PC, positive control; H&E, hematoxylin and eosin; COX-2, cyclooxygenase-2; HMGB-1, high-mobility group box-1.

H&E staining of liver sections showed portal inflammation, substantial congestion and massive amounts of dead hepatocytes in the periportal and intralobular areas in the PC group (Figure 1A). The *B. cereus* group exhibited marked improvements in hepatic degeneration and sporadic degeneration as well as much milder inflammatory infiltration.

The hepatic expression of related inflammatory cytokines was evaluated by quantitative PCR. Compared with that in the NC group, the increase of IL-13 induced by D-GalN was lower in the *B. cereus* group (*P* < 0.05) (Figure 1B). In addition, compared with those in the NC group, the levels of the anti-inflammatory cytokine IL-10 exhibited a decreasing trend after the D-GalN injection, which was improved by pretreatment with the *B. cereus* supplement (*P* < 0.05) (Figure 1B). High-mobility group box 1 (HMGB-1) exerts a detrimental effect on the pathogenesis of hepatic inflammation (Yang et al., 2004; Curtin et al., 2009; Dragomir et al., 2011). A significantly lower level of HMGB-1 was observed in the *B. cereus* group than in the PC group (*P* < 0.01) and NC group (*P* < 0.01). The expression of cyclooxygenase-2 (COX-2), a classic marker of necrosis and inflammation (Lim and Kim, 2017), was also reduced in the *B. cereus* group compared to that in the PC group (*P* < 0.05), although its expression was increased compared to that in the NC group (*P* < 0.01) (Figure 1B). Endotoxins activate hepatic macrophages via Toll-like receptors (TLRs), which trigger the hepatic migration of neutrophils and monocytes (Singh et al., 2011) and ultimately culminate in hepatic injury and systemic inflammation (Woodhouse et al., 2018). A significantly lower hepatic level of TLR-4 mRNA was detected in the probiotic group compared to that in the PC group (*P* < 0.01) (Figure 1B). Furthermore, the preventive effect on inflammation was confirmed by examining the serum levels of different cytokines.

Twenty-four hours after the D-GalN injection, e strong inflammatory responses induced by D-GalN in rats were observed in plasma, as demonstrated by notable increase cytokine levels including colony-stimulating factor (M-CSF, G-CSF, GM-CSF), pro-inflammatory cytokines (IL-1α, IL-1β, IL-2, IL-5, IL-6, IL-12, IL-17, IL-18, GRO/KC, and TNF- α), VEGF and IFN-γ (Supplementary Table 2). Compared with the D-GalN group, the *B. cereus* prophylaxis reduced the level of both IL-7 (*P* < 0.05) and RANTES (*P* < 0.05), but elevated that of the anti-inflammatory cytokine IL-10 (*P* < 0.01) to a level even higher than that of the NC group (*P* < 0.01) (Table 1). The *B. cereus* pretreatment reduced the levels of the cytokine IL-7 (*P* < 0.05). This strain improved the production of the anti-inflammatory cytokine IL-10 (*P* < 0.01) (Table 1). Plasma IL-10 levels were increased compared with those in the NC group (*P* < 0.01). Among the chemokines, the level of RANTES was normalized by the *B. cereus* pretreatment (*P* < 0.05). Thus, cytokines play major roles in inducing hepatic damage.

### *B. cereus* Decreased Plasma Endotoxin Levels, Modulated TLRs, and Reinforced Gut Barrier Function

Plasma endotoxins, which are markers of intestinal barrier function (Nier et al., 2017), are regarded as a key element of the progression of liver damage. LBP, which is strongly correlated with LPS, is used as a biological marker of bacterial infections (Muta and Takeshige, 2001). The effects of *B. cereus* on LBP are shown in Table 1. Significantly higher serum LBP levels were observed in the PC group (172.13 ± 37.8 EU/ml) than in the NC group (72.98 ± 31.75 EU/ml, *P* < 0.01). Gavage administration of *B. cereus* significantly decreased the production of LBP (*P* < 0.05). Pattern recognition receptors (PRRs) and TLRs recognize microbial LPSs to activate the innate immune response (Nakamoto et al., 2017). The mRNA expression of TLR-2 and TLR-3 was significantly increased in the *B. cereus* group compared with that in the PC group. TLRs may play a crucial role in these protective mechanisms.

Perturbations in gut integrity and permeability, a major factor associated with the disruption of tight junctions, may contribute to BT, which induces a chronic or acute inflammatory response (Nagpal and Yadav, 2017).

Compared to those in the NC group, the incidence rates of anaerobic BT increased in the livers and MLNs of animals in the PC group to 83.3% (5/6, *P* < 0.05) and 85.7% (6/6, *P* < 0.01), respectively (Table 2). Additionally, prophylaxis with *B. cereus* decreased the anaerobic BT in tissue relative to that in rats with liver injury in the PC group as follows: liver (2/7, *P <* 0.05) and venous blood (3/7, *P <* 0.05).

**TABLE 2:** Effects of *B. cereus* on BT during D-GalN-induced acute liver injury.

**Culture conditions**	**Sample**	**NC (negative control) (n/N, *N* = 7)**	**PC (positive control) (n/N, *N* = 6)**	B. cereus (n/N, N = 7)
Aerobic	Artery	3/7(42.8%)	5/6(83.3%)	4/7(57.1%)
	Vein	2/7(28.5%)	4/6(66.6%)	3/7(42.8%)
	Liver	4/7(57.1%)	6/6(100%)	4/7(57.1%)
	Kidney	3/7(42.8%)^a^	6/6(100%)	6/7(85.7%)
	MLNs	4/7(57.1%)	6/6(100%)	5/7(71.4%)
Anoxygenic	Artery	2/7(28.5%)	3/6(50%)	2/7(28.5%)
	Vein	0/7(0.00%)^b^	6/6(100%)	3/7(42.8%)^a^
	Liver	2/7(28.5%)^a^	5/6(83.3%)	2/7(28.5%)^a^
	Kidney	2/7(28.5%)	4/6(66.6%)	5/7(71.4%)
	MLNs	0/7(0.00%)^b^	6/6(85.7%)	4/7(57.1%)

**Data are presented as the mean ± SEM. ^a^P < 0.05 and ^b^P < 0.01 compared with the PC group.**

**FIGURE 2:**
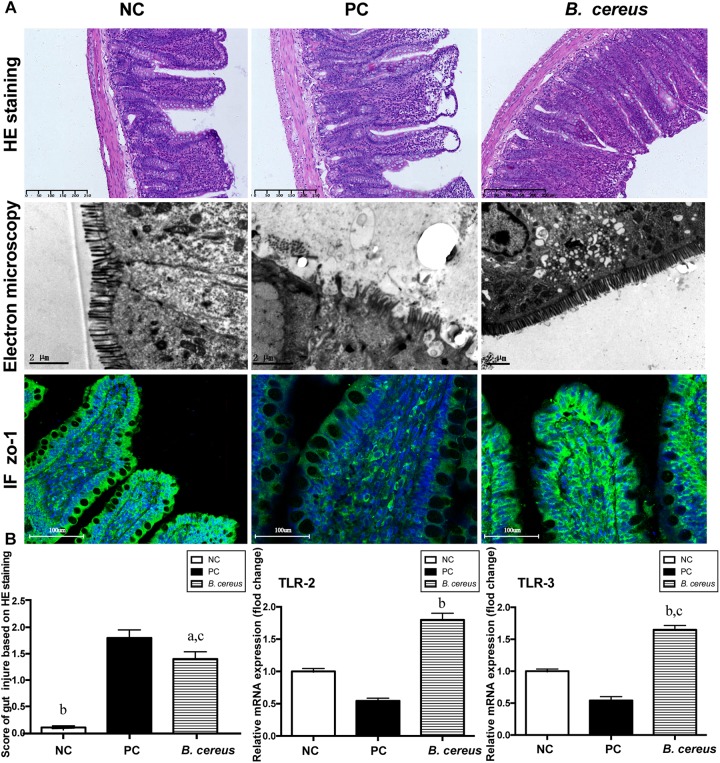
Pretreatment with *B. cereus* reinforced the intestinal barrier function. **(A)** Representative images of H&E staining of the terminal ileum; ultrastructural and histological alterations in the ileum were assessed using ZO-1 immunofluorescence (IF) staining (×20). **(B)** Ileal inflammation was monitored based on the histology scores. Ileal mRNA expression of TLR-2 and TLR-3 was determined by quantitative PCR. All data are presented as the mean ± SEM. (NC group, *n* = 7; PC group, *n* = 6; *B. cereus* group, *n* = 7). ^a^*P <* 0.05 and ^b^*P <* 0.01 compared with the PC group, ^c^*P <* 0.05 for the comparison of the *B. cereus* group with the NC group. NC, negative control; PC, positive control.

The efficacy of *B. cereus* in reinforcing the gut barrier was assessed to explore the mechanism underlying the change in BT. The integrity of the intestinal mucosa was assessed by H&E staining. As shown in Figure 2B, pretreatment with *B. cereus* alleviated the D-GalN-induced histological abnormalities in the terminal ileum, as evidenced by the lower histological scores, lower rate of subepithelial Gruenhagen’s space and better integrated villus architecture. Transmission electron microscopy was used to assess the intestinal mucosal integrity. In the PC group, the microvilli on intestinal epithelial cells (IECs) ruptured, resulting in a few stunted microvilli. After *B. cereus* supplementation, less breakdown of the brush border was observed compared to that in the PC group (Figure 2A).

Ileal tight junction proteins associated with mucosal integrity were detected using immunofluorescence staining. In the crypts and apical region of the intestinal epithelium, obvious ZO-1 protein distribution was observed in the *B. cereus* group (Figure 2A). ZO-1 staining of the ileum of the PC group revealed degradation and disorganization.

### *B. cereus* Ameliorated Alterations in the Microbiome and Reshaped the Intestinal Flora

**FIGURE 3:**
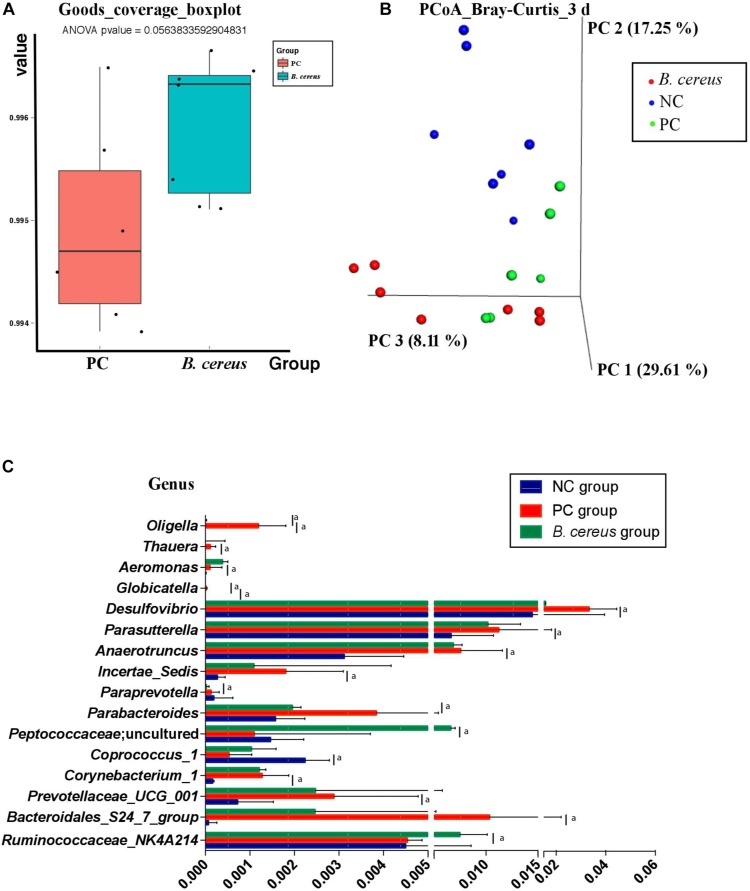
Effects of pretreatment with *Bacillus cereus* on the overall changes in the gut microbial community structure during D-GalN-induced acute liver injury. **(A)** The α-diversity of the gut microbiome determined based on the goods_coverage index of the probiotic group compared with the PC group. **(B)** The principal coordinate analysis (PCoA) plot shows the β-diversity of the gut microbiome with Bray–Curtis dissimilarity derived from 16S RNA sequencing data. **(C)** Comparison of taxa at the bacterial genus level between the PC and *B. cereus* groups. Data are presented as medians with first and third quartiles. (NC group, *n* = 7; PC group, *n* = 6; *B. cereus* group, *n* = 7). ^a^*P <* 0.05 compared with the PC group. NC, negative control; PC, positive control; PCoA, principal coordinate analysis.

The V3-V4 regions of 16S rRNA in cecal samples were sequenced to explore the change in the composition of the gut microbiota following pretreatment with *B. cereus*. Unlike that in animals of the PC group, the community richness and microbial diversity, which were calculated using the goods_coverage index, were not significantly improved in animals treated with *B. cereus* (Figure 3A). We next examined alterations in the microbial composition with principal coordinate analysis (PCoA) clustering using the Bray–Curtis index (beta diversity). The microbial composition of the *B. cereus* group on PC1 was clearly separated from that of the other two groups, explaining 29.61% of the variance (Figure 3B), showing a marked difference between the probiotic group and the two control groups. Furthermore, according to the Bray–Curtis analysis results, the microbiota of the *B. cereus* group clustered separately from those of the NC group (ANOSIM, *P* < 0.01) and PC group (ANOSIM, *P* < 0.05). The composition of the gut microbiota was clearly modulated in rats supplemented with this probiotic strain.

Regarding the taxonomic composition, at the genus level, phenotypic changes were characterized, and taxon abundance was quantitatively compared (Figure 3C). Compared to those in the NC group, samples in the PC group exhibited an enriched abundance of the common pathogens *Corynebacterium_1*, *Aeromonas*, *Bacteroidales_S24_7_group*, *Prevotellaceae_UCG_001*, *Anaerotruncus*, *Parasutterella*, and *Thauera* at the genus level. However, the abundances of these genera have not been reported to be related to liver disease.

The relative abundance of intestinal microbiota was altered after the continuous administration of *B. cereus* (Figure 3C). Bacteria-treated rats in the PC group harbored a diminished richness of *Oligella* and *Globicatella*. The pretreatment induced significant enrichment of *Peptococcaceae*; uncultured and *Ruminococcaceae* _NK4A214_group, both of which belong to the order *Firmicutes* (Figure 3C), and lower abundances of *Parabacteroides*, *Paraprevotella*, and *Desulfovibrio*.

**FIGURE 4:**
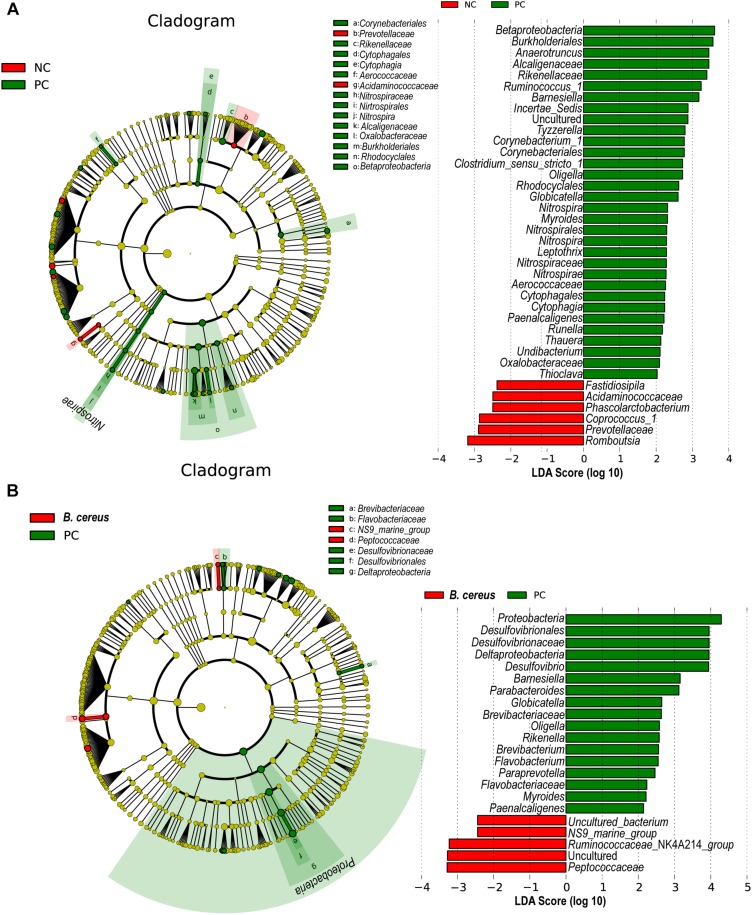
Effects of pretreatment with *B. cereus* on alterations in gut bacterial taxonomic abundance during D-GalN-induced acute liver injury. Bacterial taxa identified as differentially abundant between groups according to the LEfSe. Bacterial taxa of the probiotic group were compared with those of the PC group at different levels. Green indicates bacterial taxa with a higher abundance in the PC group, and red indicates bacterial taxa with a higher abundance in the other group. **(A)** The comparison between the NC and PC group. **(B)** The comparison between the PC and *B. cereus* group (NC group, *n* = 7; PC group, *n* = 6; *B. cereus* group, *n* = 7); NC, negative control; PC, positive control; LEfSe, linear discriminant analysis effect size.

LEfSe is used to perform linear discriminant analysis of samples according to different grouping conditions to identify communities or species displaying significant differences in separate samples and emphasizes both statistical and biological relevance. Differential abundance of biomarkers was identified among the three groups using LEfSe; in addition, biological consistency was considered (Segata et al., 2011). *B. cereus* administration significantly prevented the D-GalN-induced increases in the abundances of opportunistic pathogens in the *Betaproteobacteria*, *Globicatella*, and *Oligella* genera. The *B. cereus* group was enriched for *Peptococcaceae* and *Ruminococcaceae* _NK4A214_group (Figures 4A,B).

**FIGURE 5:**
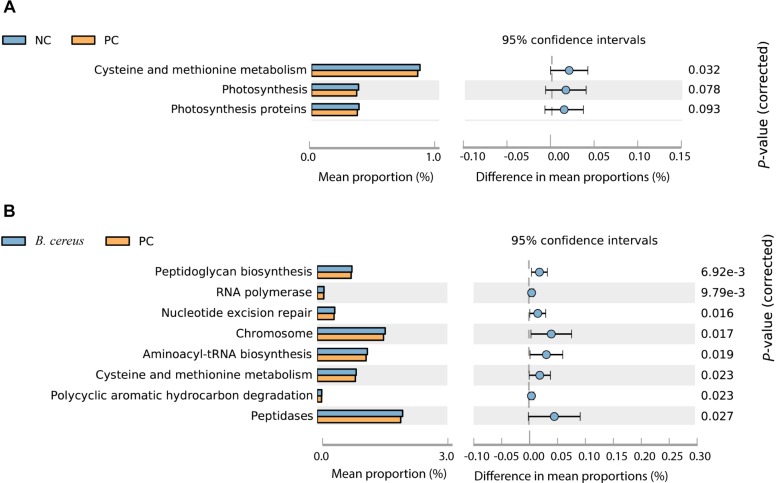
Predicted metabolic functions at KEGG level 3. NC, negative control; PC, positive control. **(A)** The comparison between the NC and PC group. **(B)** The comparison between the PC and *B. cereus* group.

Imputed bacterial KEGG pathways were used to predict significant differences in the metagenomic functions of the groups. Among the significantly different pathways, D-GalN injection inhibited cysteine and methionine metabolism (Figure 5A). Unlike that in the PC group, the metabolism of cysteine and methionine was restored in the group administered *B. cereus* (*P* = 0.023) (Figure 5B). Consistent with our results, an accumulation of L-methionine was shown to be related to the GalN concentration, suggesting that GalN induces disturbances in methionine metabolism (Ozturk et al., 1984). Additionally, the activity of glutamine synthetase was reportedly substantially reduced in mice treated with LPS/D-GalN (Jambekar et al., 2011).

Additionally, the administration of *B. cereus* affected bacterial peptidoglycan biosynthesis, RNA polymerase, nucleotide excision repair, chromosomes, aminoacyl-tRNA biosynthesis, polycyclic aromatic hydrocarbon degradation and peptidase metabolism (Figures 5A,B).

### *B. cereus* Modified the Rats’ Intestinal Flora and Affected the Host Response to D-GalN Challenge

**FIGURE 6:**
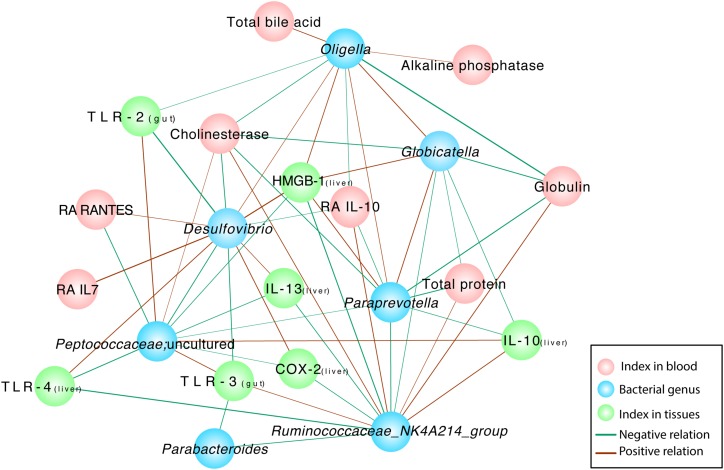
Correlation network analyses between gut bacterial genera and indexes and between different gut bacterial genera in rats with D-GalN-induced liver failure treated with/without *B. cereus*. Spearman correlation analysis was performed, and only correlations with *P* < 0.05 and *r* > 0.5 are displayed. Correlation network analyses were performed between gut bacterial genera and indexes and between different gut bacterial genera. Blue nodes represent bacterial genera, green nodes represent tissue cytokines, and red nodes represent blood indexes. “Ra” represents plasma cytokines detected using a rat cytokine assay. A red line connecting nodes represents a positive correlation, and a green line represents a negative correlation. The value of the corresponding correlation coefficient is indicated by the thickness of the line; thicker lines are correlated with higher coefficients. NC, negative control; PC, positive control.

Correlation network analysis was conducted to verify the outcomes induced by alterations in the microbiota community structure. The examination further elucidated the relationship between injury parameters and the microbial community at the genus level (Figure 6). The relative abundances of several probiotic-altered gut bacteria were associated with inflammatory cytokines and endotoxins (Figure 6). Negative correlations were identified between the microbial community *Ruminococcaceae_NK4A214_group* and *Parabacteroides* (*r* = –0.681, *P* = 0.01), *Paraprevotella* (*r* = –0.718, *P* = 0.006) and *Globicatella* (*r* = –0.635, *P* = 0.02) as well as between *Ruminococcaceae_NK4A214_group* and the levels of the inflammatory cytokines TLR-4 (*r* = –0.791, *P* = 0.001), COX-2 (*r* = –0.654, *P* = 0.015), IL-13 (*r* = –0.681, *P* = 0.01), and HMGB-1 (*r* = –0.780, *P* = 0.002). Positive correlations were observed between *Ruminococcaceae_NK4A214_group* and the TLR-3 (*r* = 0.626, *P* = 0.022), cholinesterase (*r* = 0.675, *P* = 0.011), globulin total protein (*r* = 0.705, *P* = 0.007), and anti-inflammatory Ra IL-10 levels in the blood (*r* = 0.709, *P* = 0.007) and the IL-10 levels in the liver (*r* = 0.725, *P* = 0.005). Microbial colonies are also relevant. The potentially pathogenic bacteria *Oligella*,*Paraprevotella* and *Globicatella* were positively correlated with each other (*r* = 0.631, *P* = 0.021; *r* = 0.719, *P* = 0.006; and *r* = 0.721, *P* = 0.005, respectively), and the former two were negatively correlated with the level of the anti-inflammatory cytokine Ra IL-10 in the blood (*r* = –0.599, *P* = 0.03; and *r* = –0.63, *P* = 0.021, respectively). *Desulfovibrio* was closely associated with the levels of TLRs, such as TLR-2 (*r* = –0.824, *P* = 0.001) and TLR-3 (*r* = –0.725, *P* = 0.005), similar to the previous bacteria, which were negatively correlated with the level of RA IL-10 (*r* = –0.571, *P* = 0.041). Among the various relationships, the level of the inflammatory cytokine HMGB1 was positively correlated with the pathogenic bacteria *Oligella* (*r* = 0.684, *P* = 0.01), *Globicatella* (*r* = 0.609, *P* = 0.027), *Paraprevotella* (*r* = 0.724, *P* = 0.005), and *Desulfovibrio* (*r* = 0.797, *P* = 0.001) and negatively correlated with *Peptococcaceae* (*r* = –0.654, *P* = 0.015) and *Ruminococcaceae_NK4A214_group* (*r* = –0.78, *P* = 0.002). Serum cholinesterase levels exhibited strong positive correlations with *Peptococcaceae* (*r* = 0.559, *P* = 0.047) and *Ruminococcaceae_NK4A214_group* (*r* = 0.675, *P* = 0.011) but negative correlations with the pathogenic bacteria *Oligella* (*r* = –0.649, *P* = 0.016), *Globicatella* (*r* = –0.566, *P* = 0.044), *Paraprevotella* (*r* = –0.712, *P* = 0.006), and *Desulfovibrio* (*r* = –0.702, *P* = 0.007).

## Discussion

*B. cereus* strains have been licensed as probiotics, which confer health benefits to the people (Zhu et al., 2016). *B. cereus* IP5832 (Bactisubtil) has been proposed as a pretreatment for humans (Hoa et al., 2000). *B. cereus* A05 (Biocerin), a sporulated *Bacillus* strain, has been used as a common probiotic for outpatients suffering from diarrhea (Johnson et al., 1949), which was proved to be effective at reducing diarrhea in patients under enteral nutrition (EN) and antibiotic therapy in a previous study (Soares et al., 2017). *Bacillus amyloliquefaciens* SC06 significantly reduced the hepatic injury and the levels of inflammatory cytokines in piglets, indicating the potential anti-inflammatory effect of this strain (Du et al., 2018). Furthermore, a mixture of *Bacillus* strains, including *B. sonorensis* JJY12-3, *B. paralicheniformis* JJY12-8, *B. sonorensis* JJY13-1, *B. sonorensis* JJY 13–3, and *B. sonorensis* JJY, was prepared to treat nonalcoholic steatohepatitis and nonalcoholic liver fatty liver disease (Kim et al., 2018). Similarly, *Bacillus* spp. treatment downregulated the expression of the pro-inflammatory cytokines TNFα, IL-1β, and IL-6, leading to a reduction in fat accumulation and chronic inflammation and contributing to its protective effect against chronic liver diseases (Kim et al., 2018). However, the mechanism by which *B. cereus* modulates the microbiota remains unknown, and few studies have focused on determining whether *B. cereus* ameliorates liver injury induced by D-GalN.

In our research, pretreatment with *B. cereus* alleviated liver injury by altering histological damage and transaminase activities. The production of inflammatory cytokines, including IL-13, HMGB-1, COX-2, and TLR-4, was attenuated, and the production of anti-inflammatory cytokines, including IL-10, was increased in both the liver and blood. As shown in the present study, *B. cereus* strengthened the gut barrier to some extent and reshaped the intestinal microbial community, which generated a beneficial, protective profile to reduce the susceptibility to D-GalN challenge. Pretreatment with *B. cereus* restored some damages of rats induced by D-GalN, making the health indicators of the *B. cereus* prophylaxis group be closer to NC group. However, the structure gut microbiota of the *B. cereus* prophylaxis group was different from those of the other two groups. This microbiota may be resulted from interactions among *B. cereus*, host and D-GalN, representing a microbiota set that is beneficial to the health of the host under this disease state.

D-GalN induced liver injury is characterized by significant inflammatory responses. Numerous cytokines such as interleukin family, colony-stimulating factor and chemokines formed a complex, overlapping communication network. Based on our data, significant increase of inflammatory markers as IL-1β, IL-6, and TNF-α was detected, which was consistent with previous studies and showed to be positively correlated with liver cell necrosis (Pan et al., 2016; Feng et al., 2017). Higher levels of colony stimulating factors including GM-CSF, M-CSF, and G-CSF were found in rats with D-GalN injection. G-CSF was able to increase the levels of neutrophilic granulocytes (Basu et al., 2002). M-CSF was proved to regulate tissue macrophage numbers and function (Ushach and Zlotnik, 2016). The immune regulatory potential of GM-CSF was discovered recently, especially in tissue (Becher et al., 2016).

The expression of IL-10, a well-characterized anti-inflammatory cytokine, was upregulated to prevent further liver inflammation in the probiotic group. Simultaneously, the *B. cereus* prophylaxis alleviated the levels of the pro-inflammatory cytokine IL-7 and chemokine RANTES, which mediated various biological processes, including angiogenesis and chemotaxis (Miller and Mayo, 2017). Oxidative stress is induced by D-GalN within the liver (Jin et al., 2014; Wei et al., 2014) in addition to increased expression of COX-2, which plays an essential role in inflammation (Liong et al., 2012) and produces prostaglandins and thromboxanes to aggravate hepatic injury (Huang et al., 2013). HMGB1 is predominantly secreted by inflammatory cells and potentially plays a role in regulating the pathophysiology of liver diseases (Tsung et al., 2005). As shown in our previous studies, HMGB1 levels are increased during the progression of liver injury (He et al., 2015) and activate TLRs (Park et al., 2004; Jiang et al., 2005). HMGB1, one of the best characterized damage-associated molecular patterns, signals necrosis and subsequently triggers inflammation (Scaffidi et al., 2002).

Gut-derived toxins, such as LPS, activate Kupffer cells in the liver and induce the TLR-4-mediated production of inflammatory cytokines (Guo et al., 2014). Accordingly, Kupffer cells express TLR-4 and are responsive to LPS (Su et al., 2000). Moreover, in response to stimulation with LPS, the anti-inflammatory cytokine IL-10 is secreted by Kupffer cells and decreases the production of proinflammatory cytokines (Knolle et al., 1995). Interestingly, according to Mueller et al. (2006) and Lotz et al. (2007), interleukin decreases the responsiveness of IECs to the TLR-4 ligand LPS. The same trend in cytokine levels was also observed in our experiments.

TLRs are required to maintain a healthy epithelial barrier and are involved in IgA production, epithelial cell proliferation and tight junction maintenance (Abreu, 2010). The current study discovered a gut microbiota/TLR-mediated mechanism of the leaky gut that impairs antibacterial responses in the liver (Hackstein et al., 2017). TLR-2 recognizes a wide range of ligands, including lipoteichoic acids, various proteins (such as lipoproteins and glycoproteins), zymosan, peptidoglycan, and LPSs from specific bacterial strains (Akira and Takeda, 2004; Beutler, 2007). TLR-2 is expressed by epithelial cells to protect against apoptosis and reorganization of ZO-1 in tight junctions (Gibson et al., 2008). TLR-3 appears to be abundantly expressed in the normal human small intestine and colon (Cario and Podolsky, 2000). TLR-3 is a sensor of small intestinal commensal bacteria and contributes to the anti-inflammatory mechanism (Tsuji et al., 2015). Consistent with these findings, our study observed increased TLR-2 and TLR-3 levels in the *B. cereus* group compared to those in the control group, which may reduce apoptosis and inflammation.

Microbial components and their byproducts derived from the gut disseminate through the portal vein to the liver (Balmer et al., 2014), which may contribute to the development and progression of liver diseases (Betrapally et al., 2016). Modulation of the intestinal flora has been shown to be a therapeutic strategy protecting against hepatic inflammation response to D-GalN (Wang et al., 2016). Specific components of the gut microbiota play an irreplaceable role in reducing BT (Adawi et al., 1997; Ewaschuk et al., 2007), circulating endotoxin levels and bacterially derived TLR agonists during acute liver injury (Nakamoto et al., 2017). Under *Bacillus cereus* supplementation, an enhanced gut barrier, an alleviated bacterial translocation, a reduced LBP and an enriched *Ruminococcaceae* in stool were detected. These findings are in line with those of Darnaud M’s study reporting that a significant shift in *Ruminococcaceae* enrichment was associated with less severe colitis and diminished bacterial translocation (Darnaud et al., 2018). In the clinic, *Ruminococcaceae* was shown to play a protective role in liver cirrhosis patients (Chen et al., 2015). Besides, *Ruminococcaceae* members are considered beneficial autochthonous taxa as butyrate producers and they are negatively correlated with the severity of acute-on-chronic liver failure (ACLF) (Chen et al., 2014). Spontaneous peritonitis caused by bacterial translocation from intestinal bacteria is the most common complication in patients with cirrhosis (Fernandez et al., 2002; Gomez-Hurtado et al., 2016). Therefore, the interactions of the gut bacteria and BT may reveal new therapeutic strategies by modulating the gut microbiota, thus attributing to the overall improvement of hepatic injuries (Gomez-Hurtado et al., 2016), which is worth further study in the future.

In our study, the level of the anti-inflammatory cytokine IL-10 positively correlated with the population densities of members of the *Ruminococcaceae_ NK4A214_group*, which exhibited significant negative correlations with the levels of the inflammatory cytokine HMGB-1 and the necrosis marker COX-2. *Peptococcaceae* were overrepresented in the microbiota of the *B. cereus* group; members of this family have been shown to be key short-chain fatty acid (SCFA) producers (Ye et al., 2017). An unknown genus of *Peptococcaceae* is potentially related to rat body weight (Luo et al., 2018). Bacteria-treated rats also harbored a distinctively lower abundance of opportunistic pathogens, including *Desulfovibrio*, *Paraprevotella*, and *Parabacteroides*, than the untreated rats; these pathogens may aggravate liver injury (Lv et al., 2016; Hu et al., 2018). The precise mechanism by which *B. cereus* influences hepatic inflammation and subsequently alters the gut microbiota composition requires further study.

Numerous *Bacillus* species were found in the gastrointestinal tracts of both normal humans and animals, indicating their harmlessness in normal hosts (Tam et al., 2006; Hong et al., 2009; Lefevre et al., 2017). We found oral administration caused neither of depression, diarrhea or death in rats, which is consistent with a former study (Trapecar et al., 2011). In addition, no macroscopic lesions were found by autopsy or electron microscope imaging. Body mass were measured to monitor the health of the animals and no significant differences were reported. As a probiotic supplement in humans, a safety assessment of *Bacillus species* showed good tolerance and no undesirable physiological effects on markers of liver and kidney function (Lefevre et al., 2017). Actually, foodborne infections caused by the use of probiotics are rare (Zhu et al., 2016), and diarrhea caused by *B. cereus* has not been reported in animals (Trapecar et al., 2011). Nevertheless, this study included only a very limited number of animals to investigate the conservatory mechanism of *B. cereus* pretreatment on reducing the susceptibility to hepatic damage caused by D-GalN. Experiments employing a larger sample size are still expected and urgently needed to verify the protective effect.

## Conclusion

In conclusion, oral *B. cereus* 11778 pretreatment exerted a clear probiotic effect on D-GalN-induced liver injury in rats by alleviating the inflammatory reaction, reinforcing gut barrier function and reshaping the gut microbiota. The potential molecular mechanisms underlying the probiotic properties of this bacterial strain should be completely elucidated in the future.

## Ethics Statement

This study was carried out in accordance with the recommendations of “Guidelines for Experimental Animals” of the Ministry of Science and Technology (Beijing, China). The protocol was approved by the performed in accordance with the “the Animal Care Committee of Zhejiang University School of Medicine” (permit number: 2017-591).

## Author Contributions

Y-TL, J-ZY, HX, and L-JL designed the experiments. D-QF and L-YY conducted the experiments. X-YB, L-XL, DS, and W-RW collected the data. X-WJ, Q-QW, and J-JX contributed analytical tools. K-CW and Y-ML drafted the manuscript. All authors have approved the manuscript.

## Conflict of Interest Statement

The authors declare that the research was conducted in the absence of any commercial or financial relationships that could be construed as a potential conflict of interest.
