# Supplementary material for: Pretreatment With Bacillus cereus Preserves Against D-Galactosamine-Induced Liver Injury in a Rat Model
Source: Front Microbiol. 2019 Jul 31;10:1751. doi: 10.3389/fmicb.2019.01751 (PMC6685349; doi:10.3389/fmicb.2019.01751)
Supplement: Supplementary file 1 [file Table_1.DOCX]

Supplementary Material

A Pretreatment with *Bacillus cereus* Preserves against D-galactosamine-Induced Liver Injury in a Rat Model

Ya-Ting Li^1†^, Jian-Zhong Ye^1†^, Long-Xian Lv^1†^, Hong Xu^2†^, Li-Ya Yang^1^, Xian-Wan Jiang^1^, Wen-Rui Wu^1^, Ding Shi^1^, Dai-Qiong Fang^1^, Xiao-Yuan Bian^1^, Kai-Cen Wang^1^, Qiang-Qiang Wang^1^, Jiao-Jiao Xie^1^, Yan-Meng Lu^1^, Lan-Juan Li^1^

^1^State Key Laboratory for the Diagnosis and Treatment of Infectious Diseases, The First Affiliated Hospital, School of Medicine, Zhejiang University, Hangzhou 310003, China; Collaborative Innovation Center for the Diagnosis and Treatment of Infectious Diseases, Hangzhou 310003, China

^2^Department of Orthopedics, Xiaoshan Traditional Chinese Medical Hospital, Hangzhou 310003, China

*** Correspondence:**Lan-Juan Li, PhD, Professor
[ljli@zju.edu.cn](mailto:ljli@zju.edu.cn)

^†^These authors have contributed equally to this work.

# Supplementary Figures and Tables

## Supplementary Figures

**Supplementary Table 1.** PCR primer sets used for the RT-PCR analysis of the indicated genes.

| **Gene** | **Forward Sequence (5'-3')** | **Reverse Sequence (5'-3')** |
| --- | --- | --- |
| β-actin | ACTCTGTGTGGATTGGTGGC | CGCAGCTCAGTAACAGTCCG |
| IL-10 | GAAGCTGAAGACCCTCTGGA | TGGCCTTGTAGACACCTTTG |
| IL-13 | GTATGGAGCGTGGACCTGAC | CACATCCGAGGCCTTTTGGT |
| TLR-2 | AAACTGTGTTCGTGCTTTCTG | GCGTCATTGTTCTCGTCAAA |
| TLR-3 | CTACAACAGCCTCCGCGAC | GTGAGGGGTCAAACGCTGTA |
| TLR-4 | CCCTGCCACCATTTACAGTT | ATCAGAGTCCCAGCCAGATG |
| COX-2 | CTCAGCCATGCAGCAAATCC | GGGTGGGCTTCAGCAGTAAT |
| HMGB-1 | GGCGGCTGTTTTGTTGACAT | ACCCAAAATGGGCAAAAGCA |

COX-2: cyclooxygenase-2; HMGB-1: high-mobility group box- 1; TLR: toll-like receptor

**Supplementary Table 2.** Effects of *B. cereus* on plasma cytokine levels.

|  | **NC**  **(negative control)**  ***N* = 7** | **PC**  **(positive control)**  ***N* = 6** | ***Bacillus cereus***  ***N* = 7** |
| --- | --- | --- | --- |
| IL-1a | 177.20 ± 13.29 ^b^ | 288.18± 19.32 | 273.41± 21.98 ^d^ |
| IL-1b | 182.03 ± 20.16 ^b^ | 371.38 ± 25.14 | 363.29 ± 34.15 ^d^ |
| IL-2 | 1220.95 ± 85.97 ^b^ | 1613.98 ± 75.01 | 1503.27 ± 97.93 |
| IL-5 | 462.50 ± 27.42 ^b^ | 630.88 ± 18.79 | 604.09 ± 41.05 ^d^ |
| IL-6 | 240.03 ± 28.78 ^b^ | 529.04 ± 46.45 | 477.96 ± 59.80 ^c^ |
| IL-12(p70) | 93.90 ± 14.51 ^b^ | 174.93 ± 13.06 | 142.50 ± 17.92 |
| IL-17 | 71.12 ± 7.54 ^b^ | 132.39 ± 8.89 | 133.11 ± 8.31 ^d^ |
| IL-18 | 1273.78 ± 83.38^a^ | 1773.49 ± 92.36 | 1668.99 ± 151.83 |
| GRO/KC | 207.53 ± 12.53 ^b^ | 379.10 ± 40.57 | 350.24 ± 24.24 ^d^ |
| TNF-a | 86.33 ± 4.86 ^a^ | 146.98 ± 16.38 | 159.56 ± 8.60 ^d^ |
| VEGF | 22.01 ± 1.74 ^b^ | 36.61 ± 2.48 | 33.16 ± 2.74 ^c^ |
| G-CSF | 12.54 ± 1.66 ^b^ | 26.58 ± 2.09 | 23.58 ± 2.44 ^d^ |
| GM-CSF | 78.73 ± 9.87 ^b^ | 164.54 ± 15.90 | 120.47 ± 16.55 |
| M-CSF | 551.32 ± 14.71 ^b^ | 858.01 ± 43.68 | 751.59 ± 66.71 ^c^ |
| IFN- γ | 185.08 ± 27.22 ^b^ | 430.03 ± 31.16 | 334.07 ± 30.91 ^c^ |

Data are presented as means ± SEM. ^a^*P* < 0.05 and ^b^*P* < 0.01 compared with the PC group, ^c^*P* < 0.05 and ^d^*P* < 0.01 for the comparison of the *B. cereus* group with the NC group. NC: negative control; PC: positive control; TNF-α: tumor necrosis factor; GRO/KC: granulocyte colony-stimulating factor; VEGF: vascular endothelial growth factor; growth-regulated oncogene-keratinocyte chemoattractant; IFN-γ: interferon; monocyte and M-CSF: macrophage colony-stimulating factor; G-CSF: granulocyte-macrophage colony-stimulating factor GM-CSF: granulocyte-macrophage colony-stimulating factor.

**Supplementary Fig 1. Rat body weight since the first day of gavage administration.** Data are shown as the mean ± SEM (n = 6-7 per group). Data are presented as means ± SEM. ^a^*P* < 0.05 and ^b^*P* < 0.01 compared with the PC group, ^c^*P* < 0.05 and ^d^*P* < 0.01 for the comparison of the *B. cereus* group with the NC group. NC: negative control; PC: positive control;
